# Smoking, Menthol Cigarettes and All-Cause, Cancer and Cardiovascular Mortality: Evidence from the National Health and Nutrition Examination Survey (NHANES) and a Meta-Analysis

**DOI:** 10.1371/journal.pone.0077941

**Published:** 2013-10-25

**Authors:** Miranda R. Jones, Maria Tellez-Plaza, Ana Navas-Acien

**Affiliations:** 1 Department of Epidemiology, Johns Hopkins University Bloomberg School of Public Health, Baltimore, Maryland, United States of America; 2 Department of Welch Center for Prevention, Epidemiology and Clinical Research, Johns Hopkins University Bloomberg School of Public Health, Baltimore, Maryland, United States of America; 3 Department of Environmental Health Sciences, Johns Hopkins University Bloomberg School of Public Health, Baltimore, Maryland, United States of America; 4 Fundacion de Investigacion del Hospital Clinico de Valencia-INCLIVA, Valencia, Spain; 5 Department of Oncology, Johns Hopkins School of Medicine, Baltimore, Maryland, United States of America; Geisel School of Medicine at Dartmouth College, United States of America

## Abstract

**Background:**

The U.S. Food and Drug Administration has the authority to regulate tobacco product constituents, including menthol, if the scientific evidence indicates harm. Few studies, however, have evaluated the health effects of menthol cigarette use.

**Objective:**

To investigate associations of cigarette smoking and menthol cigarette use with all-cause, cancer and cardiovascular risk in U.S. adults.

**Methods:**

We studied 10,289 adults ≥ 20 years of age who participated in the National Health and Nutrition Examination Survey from 1999-2004 and were followed through December 2006. We also identified studies comparing risk of all-cause mortality, cardiovascular disease and cancer for menthol and nonmenthol cigarette smokers and estimates were pooled using random-effects models.

**Results:**

Fifty-five percent of participants were never smokers compared to 23%, 17% and 5% of former, current nonmenthol and current menthol cigarette smokers, respectively. The adjusted hazard ratios (95% CI) for former, current nonmenthol and current menthol cigarette smokers compared to never smokers were 1.24 (0.96, 1.62), 2.40 (1.56, 3.71) and 2.07 (1.20, 3.58), respectively, for all-cause mortality; 0.92 (0.62, 1.37), 2.10 (1.02, 4.31) and 3.48 (1.52, 7.99) for cardiovascular mortality; and 1.91 (1.21, 3.00), 3.82 (2.19, 6.68) and 2.03 (1.00, 4.13) for cancer mortality. Using data from 3 studies of all-cause mortality, 5 of cardiovascular disease and 13 of cancer, the pooled relative risks (95% CI) comparing menthol cigarette smokers to nonmenthol cigarette smokers was 0.94 (0.85, 1.05) for all-cause mortality, 1.28 (0.91, 1.80) for cardiovascular disease and 0.84 (0.76, 0.92) for any cancer.

**Conclusions:**

In a representative sample of U.S. adults, menthol cigarette smoking was associated with increased all-cause, cardiovascular and cancer mortality with no differences compared to nonmenthol cigarettes. In the systematic review, menthol cigarette use was associated with inverse risk of cancer compared to nonmenthol cigarette use with some evidence of an increased risk for cardiovascular disease.

## Introduction

Tobacco use is the leading cause of preventable mortality in the United States[1]. The burden of tobacco-related disease, however, is not uniformly distributed across the population. African Americans have the highest mortality rates for coronary heart disease and stroke followed by non-Hispanic Whites and Hispanics, respectively [2–4]. For all cancer sites combined, African American men and women have a higher mortality rate compared to their White counterparts [4–6]. Racial differences in disease burden remain unexplained even after adjustment for clinical and health care risk factors, which has lead to the exploration of other potential explanations for these disparities. It has been hypothesized that menthol cigarette use, which is highly prevalent in African-American smokers, may contribute to these disparities. Mentholated cigarettes may pose a relatively greater health risk because the cooling and anti-irritant effects of menthol may facilitate smoking initiation [7,8], reduce cessation [9–15] or could result in deeper inhalation and absorption of harmful tobacco toxicants [16–19], although findings in these areas have been mixed.

The U.S. Food and Drug Administration has the authority to regulate tobacco product constituents, including the use of menthol, if the scientific evidence indicates harm. Findings from studies evaluating differences in chronic health effects between smoking menthol and nonmenthol cigarettes have been mixed. Overall, little is known about the impact of menthol cigarette smoking compared to regular cigarettes in total and cause-specific mortality. The objective of this study was to investigate the prospective association of cigarette smoking and menthol cigarette use with all cause, cardiovascular and cancer mortality in U.S. adults who participated in the National Health and Nutrition Examination Survey (NHANES) from 1999 through 2004 and were followed through December 31, 2006. We also performed a meta-analysis to quantitatively summarize the epidemiologic evidence concerning menthol cigarette use and all cause, cardiovascular and cancer endpoints.

## Methods

### Study Population

NHANES is conducted by the U.S. National Center for Health Statistics (NCHS; Centers for Disease Control and Prevention [CDC], Atlanta, GA), using a complex multistage sampling design to obtain a representative sample of the civilian non-institutionalized U.S. population. Data come from publically-available 1999- 2004 NHANES, which can be accessed at http://www.cdc.gov/nchs/nhanes.htm. NHANES study protocols for the 1999-2004 survey years were approved by the National Center for Health Statistics Institutional Review Board, and oral and written informed consent was obtained from all participants. NCHS ensures that the identity of the participants is not disclosed and all direct identifiers, as well as any characteristics that might lead to identification, were omitted from the linked dataset used in the present study. 

For this analysis we used data from 15,332 adults 20 years of age and older who participated in the NHANES 1999-2004 interviews and examinations. We excluded 21 participants ineligible for mortality follow-up (insufficient data for matching), 832 pregnant women, 36 participants missing information on smoking status, 1,536 participants missing body mass index and 1,595 participants missing other relevant covariates. We further excluded 105 current smokers who were missing information on cigarette type (menthol vs. nonmenthol), 916 former and current smokers missing information on years of smoking (data needed to estimate pack-years of smoking), and 2 participants who died the same year as their NHANES examination, leaving 10,289 participants for this study. Sociodemographic characteristics of study participants were comparable to overall NHANES 1999-2004 population (data not shown). The overall participation rate of adults ≥20 years of age in NHANES examinations was 70% for survey years 1999-2004.

### Cigarette smoking status and menthol cigarette use

 Information on participant smoking status and behavior was obtained from a self- reported questionnaire. Participants were classified as never smokers if they had not smoked at least 100 cigarettes in their lifetime. Former smokers were defined as individuals who had smoked 100 cigarettes in their lifetime but were not currently smoking. For participants who reported current smoking, cigarette type was determined by the brand that they smoked at the time of the interview and categorized as menthol or nonmenthol. Cumulative pack-years of smoking were calculated using the self-reported number of cigarettes smoked per day in the past five days for current smokers (or before quitting for former smokers) and the number of years of smoking. 

### Mortality follow-up

Participants were followed for mortality from the date of NHANES survey participation (1999-2004) through December 31, 2006. Vital status and cause of death were determined by probabilistic matching between NHANES records and death certificates from the National Death Index [20]. The cause of death was determined using the underlying cause listed on death certificates, and was coded using the International Classification of Diseases, 10^th^ Revision [21]. Cause-specific mortality was ascertained for cardiovascular disease (codes I00-I78), heart disease (codes I00-I09, I11, I13, I20-I51), any cancer (codes C00-C97) and smoking-related cancers (codes C00-C14 [lip, oral cavity, pharynx], C15 [esophagus], C16 [stomach], C18-C21 [colon, rectum], C22 [liver], C25 [pancreas], C32 [larynx], C33-C34 [trachea, bronchus, lung], C53 [cervix], C64-C65 [kidney], C67 [bladder]). We could not evaluate stroke mortality or cancer specific mortality (e.g. lung cancer) due the small number of deaths for those endpoints. Follow-up time for each participant was calculated as the difference between the age at the date of the NHANES examination and the age at the date of death or end of the mortality follow-up period (December 31, 2006), whichever occurred first. 

### Other Variables

Information on sex, age, race/ethnicity and education was collected by self-reported questionnaire. Race/ethnicity was subsequently categorized by NCHS as non-Hispanic white, non-Hispanic black, Mexican-American, other Hispanic, and other. Body mass index (BMI) was calculated by dividing measured weight in kilograms by measured height in meters squared. Three (and in some cases four) systolic and diastolic blood pressures were measured on the same day in a sitting position. Hypertension was defined as a mean systolic blood pressure ≥ 140 mmHg, a mean diastolic blood pressure ≥ 90 mmHg, a self-reported physician diagnosis, or use of antihypertensive medication. Diabetes was defined as a fasting serum glucose ≥ 126 mg/dL, a nonfasting serum glucose ≥ 200 mg/dL, a self-reported physician diagnosis, or medication use. Serum total cholesterol was measured enzymatically. High density lipoprotein (HDL) cholesterol was measured by heparin-manganese precipitation for NHANES 1999- 2002 and by direct immunoassay for NHANES 2003-2004. Serum creatinine was measured by a kinetic rate Jaffé method and was calibrated to account for laboratory differences across survey years [22]. Estimated glomerular filtration rate was calculated from calibrated serum creatinine, age, sex and race/ethnicity by using the Modification of Diet in Renal Disease Study formula[22,23]. Cadmium was measured in whole blood on a PerkinElmer Model SIMAA 6000 multielement atomic absorption spectrometer, with Zeeman background correction in 1999–2002 and on an inductively coupled plasma-mass spectrometer in 2003–2004. The limit of detection for blood cadmium was 0.3 μg/L for survey years 1999–2002 and 0.2 μg/L for survey years 2003–2004. For participants with cadmium concentrations below the limits of detection (N= 484), a level equal to the limit of detection divided by the square root of two was assigned.

### Statistical Analysis

We estimated crude and multivariable adjusted hazard ratios (95% confidence intervals) for mortality end-points using Cox proportional hazards regression with age as time scale and individual starting follow-up times (age at examination) treated as staggered entries. Initially we adjusted statistical models for sex, race/ethnicity (White/African-American/Mexican-American/Other) and education (<high school/high school/high school) (model 1). Second, we further adjusted for body mass index (continuous), total cholesterol (continuous), HDL cholesterol (continuous), cholesterol-lowering medication use (yes/no), hypertension (yes/no), antihypertensive medication use (yes/no), diabetes (yes/no) and estimated glomerular filtration rate (eGFR) (continuous) (model 2). To evaluate potential differences in the association between menthol cigarette use and mortality by differences in the duration and intensity of smoking, we further adjusted for pack-years of smoking (continuous) (model 3). In previous work in NHANES 1999- 2010, current menthol cigarette smokers were found to have higher concentrations of blood cadmium, a highly toxic and carcinogenic tobacco constituent [19]. We therefore further adjusted models for blood cadmium concentrations (log-transformed) and estimated the percent attenuation in the hazard ratios in order to evaluate the potential influence of blood cadmium, on difference in mortality risk (model 4). Due to substantial missing data on household income (N=1,013), education was used as the primary measure of socioeconomic status; to evaluate the potential influence of income we conducted a sensitivity analysis further adjusting for the poverty-to-income ratio (PIR; the ratio of the midpoint of the family’s income category to its appropriate poverty threshold as defined by the US Census Bureau) in 9,445 participants with available data for this measure. PIR was categorized as low (PIR ≤ 1.30), medium (PIR 1.31- 3.50) and high (PIR>3.50) [24]. All statistical analyses were performed using the survey package (version 3.23) in R software [25,26] (version 2.12.1) to account for the complex sampling design and weights in NHANES 1999-2004 and to obtain appropriate estimates and standard errors. All statistical tests were 2-sided and confidence intervals were set at 95%.

### Meta-analysis

**Figure 1:**
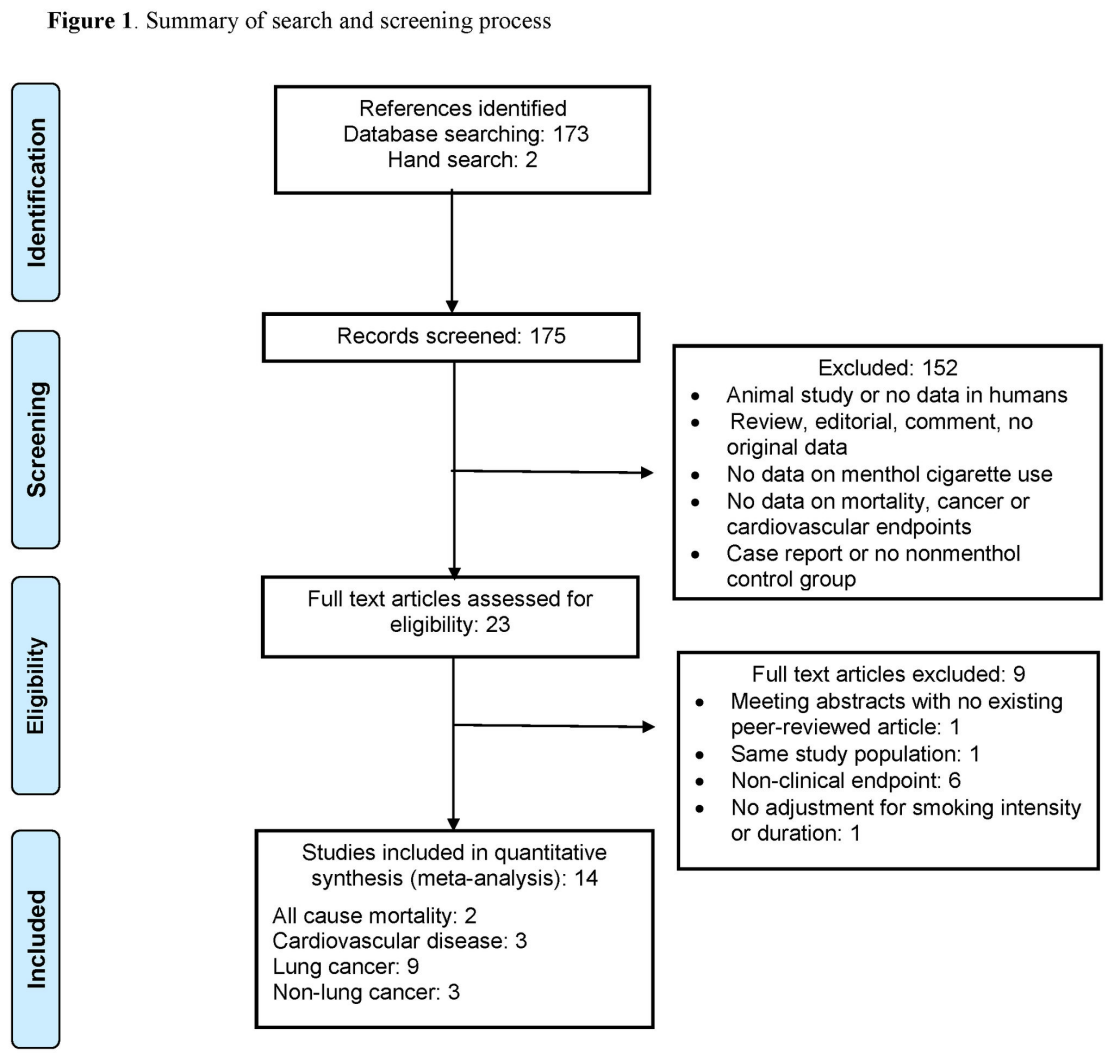
Summary of search and screening process.

To further summarize the potential relationship between menthol cigarette use and health outcomes, we conducted a systematic review of epidemiologic studies that have investigated the association between menthol cigarette use and all-cause mortality, cancer or cardiovascular disease outcomes. We searched PubMed for relevant published studies from the beginning of indexing through January 2013 using the following combination of Medical Subject Heading (MeSH) terms and text words: Menthol*[all fields] AND ("Tobacco"[Mesh] OR "Smoking"[Mesh] OR Tobacco[all fields]) AND (Health[all fields] OR Mortality[all fields] OR Cancer[all fields] OR "Cardiovascular Diseases"[Mesh] OR Cardiovascular[all fields]). The search strategy retrieved 173 citations. Two investigators (M.R.J. and M.T-P) independently reviewed each paper and applied the study selection criteria (Figure 1). An important inclusion criterion was the requirement of adjustment for differences in smoking intensity or duration. After retrieval of articles from the search, the reference lists of selected articles were checked for other potentially relevant articles. Relative risk estimates comparing menthol to nonmenthol cigarette use were extracted from included studies. For each outcome (all cause mortality, lung cancer, other cancers, overall cancer and cardiovascular disease), we pooled estimates from individual studies and the results of the current analysis using a random effects model. For studies that reported estimates stratified by subgroups (men and women, White and African-American) [27–32] an overall estimate was derived for the study by pooling the stratified estimates. Heterogeneity across studies was evaluated using the I^2^ statistic [33]. For each outcome, we also examined the relative influence of each study on pooled estimates by omitting one study at a time. Finally, we assessed publication bias using funnel plots. The meta-analysis was performed using STATA software, version 11.2.

## Results

### Menthol cigarette use in NHANES

A total of 1395 (16.8%) participants smoked nonmenthol cigarettes and 627 (5.4%) participants smoked menthol cigarettes. A total of 8267 participants were non-smokers (54.9% never smokers and 22.9% former smokers). Current smokers (menthol and nonmenthol cigarettes) were similar with regard to risk factors such as age, education, body mass index, eGFR, diabetes status and cholesterol levels (Table 1). Participants who currently smoke menthol cigarettes were more likely to be female, African American, to have hypertension and to have smoked fewer pack-years compared to current nonmenthol cigarette smokers. Menthol cigarette smokers also had higher blood cadmium concentrations compared to smokers of nonmenthol cigarettes. 

**Table 1:** Participant Characteristics by Smoking Status.

	Current smokers		Non-smokers	
Characteristics	Nonmenthol	Menthol	Never smokers	Former smokers
Overall	1395 (16.8)	627 (5.4)	5710 (54.9)	2557 (22.9)
Sex				
Male	855 (58.0)	301 (41.2)	2352 (42.6)	1588 (57.8)
Female	540 (42.0)	326 (58.8)	3358 (57.4)	969 (42.2)
Age, yr	41.5 (0.4)	42.0 (0.6)	45.2 (0.4)	55.4 (0.4)
Race/ethnicity				
White	892 (81.6)	193 (51.6)	2659 (69.3)	1568 (81.6)
African-American	109 (3.1)	365 (38.2)	1142 (11.5)	317 (5.7)
Mexican-American	278 (5.6)	32 (1.4)	1406 (8.0)	527 (5.3)
Other	116 (9.7)	37 (8.9)	503 (11.2)	145 (7.3)
Education				
<High school	525 (26.9)	222 (29.4)	1695 (16.5)	828 (19.5)
High school	396 (34.5)	206 (35.7)	1241 (22.6)	596 (25.7)
High school	474 (38.7)	199 (34.9)	2774 (60.8)	1133 (54.8)
Pack-years of smoking	23.9 (0.7)	19.6 (1.0)	0 (0)	22.8 (0.7)
Blood cadmium, μg/L	0.99 (0.95, 1.03)	1.04 (0.97, 1.12)	0.30 (0.29, 0.31)	0.40 (0.38, 0.41)
Body mass index, kg/m^2^	26.9 (0.2)	27.9 (0.4)	28.2 (0.2)	28.8 (0.2)
Medication use				
Antihypertensive	208 (11.0)	116 (14.5)	1332 (18.5)	888 (28.9)
Cholesterol-lowering	116 (7.1)	44 (6.7)	618 (9.4)	539 (18.9)
eGFR, ml/min/1.73m2	98.4 (1.0)	100.4 (1.4)	94.2 (0.7)	86.5 (0.7)
Total cholesterol	203.9 (1.3)	198.1 (2.2)	200.8 (0.6)	208.1 (1.4)
HDL cholesterol	48.3 (0.6)	51.8 (0.7)	52.9 (0.3)	52.5 (0.5)
Hypertension				
Yes	438 (25.4)	239 (33.9)	2372 (34.9)	1430 (48.8)
No	957 (74.6)	388 (66.1)	3338 (65.1)	1127 (51.2)
Diabetes				
Yes	135 (6.6)	59 (7.8)	608 (7.2)	417 (11.4)
No	1260 (93.4)	568 (92.2)	5102 (92.8)	2140 (88.6)
Prior history				
Cardiovascular disease	35 (2.2)	6 (0.6)	151 (1.9)	125 (3.7)
Cancer	27 (2.0)	9 (1.8)	108 (1.7)	101 (3.4)
Respiratory disease	74 (6.6)	27 (5.4)	238 (4.3)	150 (6.6)

Values represent No. (weighted %) for categorical variables or means (standard errors) for continuous variables, except for blood cadmium for which geometric means (95% CI) are reported

The mean follow-up time was 4.8 years for participants who were alive at the end of follow-up, and 3.3 years for participants who died before the end of follow-up. The numbers of deaths due to all causes, cardiovascular disease, heart disease and cancer were 635, 225, 137 and 153, respectively. The multivariable adjusted hazard ratios (95% confidence intervals) for former, current nonmenthol cigarette smokers and current menthol cigarette smokers compared to never smokers were, respectively, 1.24 (0.96, 1.62), 2.40 (1.56, 3.71) and 2.07 (1.20, 3.58) for all-cause mortality; 0.92 (0.62, 1.37), 2.10 (1.02, 4.31) and 3.48 (1.52, 7.99) for cardiovascular mortality; 1.46 (0.83, 2.56), 4.18 (1.99, 8.79) and 5.39 (1.87, 15.54) for heart disease mortality; and 1.91 (1.21, 3.00), 3.82 (2.19, 6.68) and 2.03 (1.00, 4.13) for cancer mortality (Table 2, Model 3). Further adjustment for the PIR (available for 9,445 participants) changed hazard ratios (95% confidence intervals) to 1.27 (0.98, 1.64), 2.37 (1.46, 3.84) and 1.96 (1.04, 3.69), for former, current nonmenthol and current menthol cigarette smokers compared to never smokers respectively, for all-cause mortality; 0.84 (0.56, 1.27), 2.21 (1.11, 4.43) and 3.63 (1.49, 8.89) for cardiovascular mortality; and 2.15 (1.37, 3.38), 4.08 (2.19, 7.60) and 2.18 (0.97, 4.90) for cancer mortality. Comparing former, current nonmenthol cigarette smokers and current menthol cigarette smokers to never smokers, further adjustment for blood cadmium concentrations attenuated the hazard ratios by 7.3%, 31.3% and 34.3% for all-cause mortality; 7.6%, 34.3% and 36.5% for cardiovascular mortality; 6.8%, 28.9% and 28.4% for heart disease mortality; and 5.8%, 25.1% and 28.1% for cancer mortality (Table 2, Model 4). 

**Table 2:** Hazard ratio (95% confidence interval) of mortality endpoints by smoking status.

	No. of deaths	Model 1	Model 2	Model 3	Model 4
**All-cause**					
Never	262	1.00 (ref)	1.00 (ref)	1.00 (ref)	1.00 (ref)
Former	255	1.55 (1.21, 1.98)	1.56 (1.24, 1.97)	1.24 (0.96, 1.62)	1.15 (0.88, 1.50)
Current					
Nonmenthol	86	3.16 (2.04, 4.90)	3.18 (2.04, 4.96)	2.40 (1.56, 3.71)	1.65 (1.04, 2.60)
Menthol	32	2.46 (1.40, 4.33)	2.62 (1.50, 4.59)	2.07 (1.20, 3.58)	1.36 (0.75, 2.48)
**Cardiovascular disease**					
Never	98	1.00 (ref)	1.00 (ref)	1.00 (ref)	1.00 (ref)
Former	91	1.15 (0.83, 1.60)	1.14 (0.85, 1.55)	0.92 (0.62, 1.37)	0.85 (0.56, 1.28)
Current					
Nonmenthol	23	2.48 (1.29, 4.77)	2.72 (1.36, 5.44)	2.10 (1.02, 4.31)	1.38 (0.58, 3.26)
Menthol	13	3.76 (1.53, 9.27)	4.36 (1.79, 10.61)	3.48 (1.52, 7.99)	2.21 (0.94, 5.20)
**Heart disease**					
Never	51	1.00 (ref)	1.00 (ref)	1.00 (ref)	1.00 (ref)
Former	62	1.75 (1.14, 2.69)	1.66 (1.07, 2.57)	1.46 (0.83, 2.56)	1.36 (0.78, 2.40)
Current					
Nonmenthol	16	4.27 (2.14, 8.50)	4.92 (2.51, 9.64)	4.18 (1.99, 8.79)	2.97 (1.41, 6.25)
Menthol	8	5.33 (1.64, 17.33)	6.25 (2.15, 18.23)	5.39 (1.87, 15.54)	3.86 (1.49, 10.01)
**Cancer**					
Never	48	1.00 (ref)	1.00 (ref)	1.00 (ref)	1.00 (ref)
Former	64	2.32 (1.50, 3.60)	2.38 (1.53, 3.69)	1.91 (1.21, 3.00)	1.80 (1.13, 2.87)
Current					
Nonmenthol	33	5.20 (2.96, 9.13)	5.11 (2.89, 9.01)	3.82 (2.19, 6.68)	2.86 (1.37, 5.97)
Menthol	8	2.58 (1.27, 5.23)	2.59 (1.28, 5.25)	2.03 (1.00, 4.13)	1.46 (0.66, 3.23)

Model 1 adjusted for sex, age, race/ethnicity, education and body mass indexModel 2 further adjusted for total cholesterol, high density lipoprotein cholesterol, cholesterol-lowering medication use, hypertension, antihypertensive medication use, diabetes and estimated glomerular filtration rate Model 3 further adjusted for pack-years of smoking

Model 4 further adjusted for blood cadmium

Comparing current menthol vs. nonmenthol cigarette use, the hazard ratios (95% confidence intervals) were 0.88 (0.54, 1.43) for all-cause mortality, 1.65 (0.73, 3.72) for cardiovascular mortality and 1.32 (0.50, 3.52) for heart disease mortality (Table 3, Model 3). For any cancer and smoking-related cancer mortality the corresponding hazard ratios were 0.57 (0.28, 1.17) and0.47 (0.18, 1.23), respectively. Further adjustment for blood cadmium concentrations, resulted in practically no change in the association between menthol cigarette use with cardiovascular disease, heart disease, overall cancer and all-cause mortality compared to nonmenthol cigarette use. 

**Table 3:** Hazard ratio (95% confidence interval) of mortality endpoints by cigarette type.

	No. of deaths	Model 1	Model 2	Model 3	Model 4
**All-cause**					
Nonmenthol	86	1.00 (ref)	1.00 (ref)	1.00 (ref)	1.00 (ref)
Menthol	32	0.80 (0.48, 1.32)	0.84 (0.52, 1.37)	0.88 (0.54, 1.43)	0.83 (0.52, 1.35)
**Cardiovascular disease**					
Nonmenthol	23	1.00 (ref)	1.00 (ref)	1.00 (ref)	1.00 (ref)
Menthol	13	1.52 (0.67, 3.48)	1.61 (0.72, 3.58)	1.65 (0.73, 3.72)	1.59 (0.72, 3.51)
**Heart disease**					
Nonmenthol	16	1.00 (ref)	1.00 (ref)	1.00 (ref)	1.00 (ref)
Menthol	8	1.28 (0.47, 3.45)	1.29 (0.50, 3.33)	1.32 (0.50, 3.52)	1.32 (0.52, 3.40)
**Cancer**					
Nonmenthol	33	1.00 (ref)	1.00 (ref)	1.00 (ref)	1.00 (ref)
Menthol	8	0.52 (0.24, 1.10)	0.53 (0.25, 1.10)	0.57 (0.28, 1.17)	0.54 (0.27, 1.07)
**Smoking-related cancer**					
Nonmenthol	28	1.00 (ref)	1.00 (ref)	1.00 (ref)	1.00 (ref)
Menthol	6	0.42 (0.16, 1.11)	0.44 (0.17, 1.14)	0.47 (0.18, 1.23)	0.41 (0.17, 0.99)

Model 1 adjusted for sex, age, race/ethnicity, education and body mass index

Model 2 further adjusted for total cholesterol, high density lipoprotein cholesterol, cholesterol-lowering medication use, hypertension, antihypertensive medication use, diabetes and estimated glomerular filtration rate

Model 3 further adjusted for pack-years of smoking

Model 4 further adjusted for blood cadmium

### Meta-analysis

**Figure 2:**
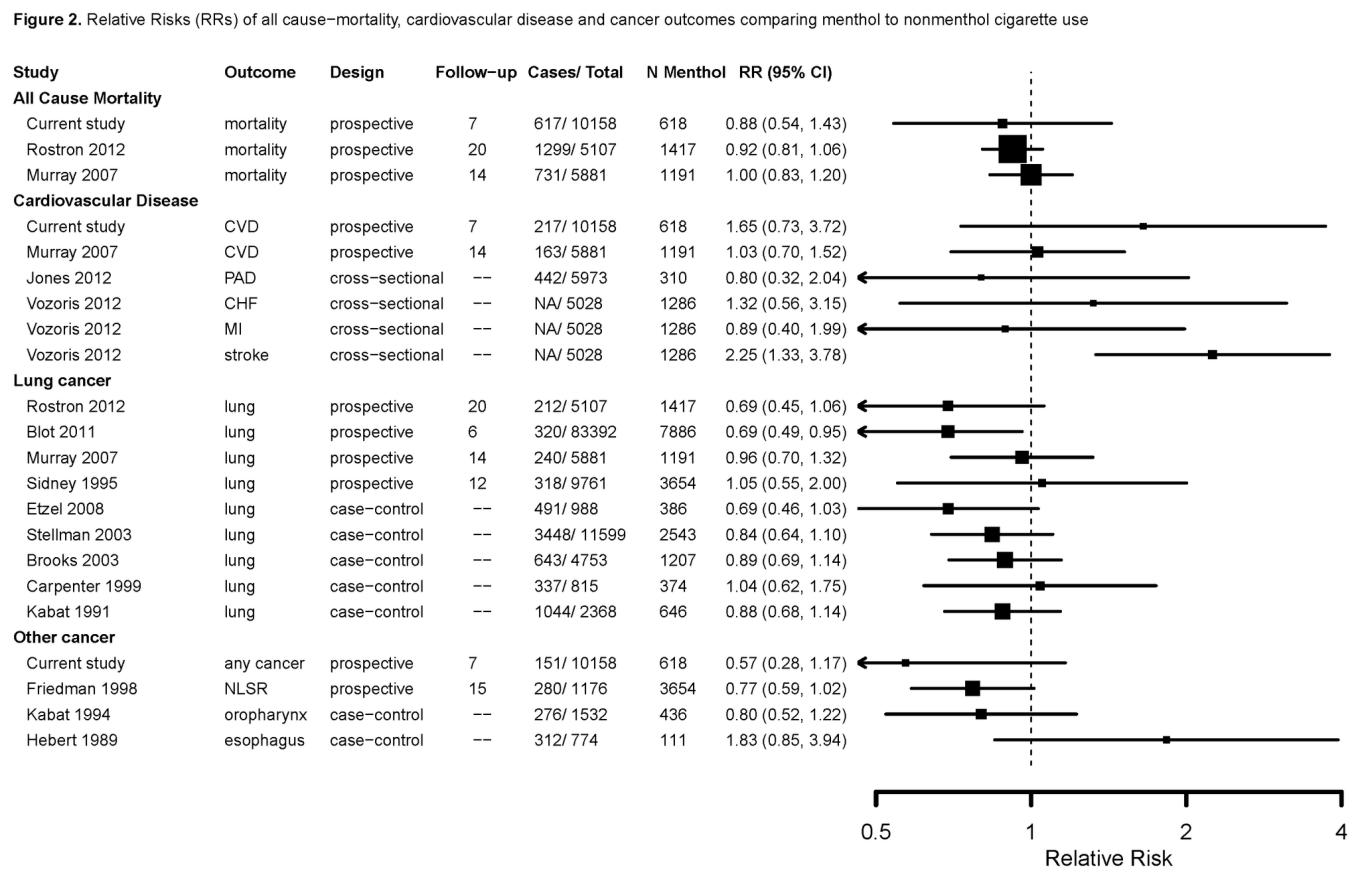
Relative Risks (RRs) of all-cause mortality, cardiovascular and cancer outcomes comparing menthol to nonmenthol cigarette use. The area of each square is proportional to the inverse of the variance of the estimated log RR. Horizontal lines represent 95% confidence intervals (CI). Follow-up represents the length of follow-up (years). Abbreviations: NA, Not Available; NLSR, Non-lung smoking-related; CVD, Cardiovascular disease; PAD, Peripheral artery disease; CHF, Congestive heart disease; MI, Myocardial infarction.

We identified 14 studies published between 1989 and 2012 for the meta-analysis (Figure 1), including 2 studies of all cause mortality [34,35], 3 of cardiovascular disease [14,34,36,37], 9 of lung cancer [29,31,32,34,35,38–41] and 3 of non-lung cancers [27,28,30]. Figure 2 summarizes the study characteristics and relative risk estimates for studies comparing risk for all-cause mortality, cardiovascular disease and cancer among menthol cigarette smokers compared to smokers of nonmenthol cigarettes. For the association between menthol cigarette use and all cause mortality, the pooled relative risk was 0.94 (95% CI: 0.85, 1.05; P for heterogeneity= 0.74; I^2^= 0.0%). For the association with cardiovascular disease, the pooled relative risk was 1.28 (95% CI: 0.91, 1.80; P for heterogeneity= 0.17; I^2^= 36.2%). Comparing menthol to nonmenthol cigarette smokers for cancer outcomes the pooled relative risk were 0.84 (95% CI: 0.76, 0.92; P for heterogeneity= 0.55; I^2^= 0.0%) for any cancer, 0.84 (95% CI: 0.75, 0.94; P for heterogeneity= 0.74; I^2^= 0.0%) for lung cancer and 0.84 (95% CI: 0.60, 1.16; P for heterogeneity= 0.15; I^2^= 44.5%) for other cancers. In the influence analysis, there was minimal change in the pooled relative risks, and there was no change in the direction of effects, when any one study was excluded (data not shown). Funnel plots did not suggest the presence of publication biases (data not shown).

## Discussion

In a representative sample of U.S. adults, current cigarette use was prospectively associated with all-cause, cardiovascular and cancer mortality with no difference in risk between current menthol and nonmenthol cigarette smokers, except maybe an increased risk for cardiovascular disease and an inverse risk of cancer mortality. For former cigarette use we had no information on menthol vs. nonmenthol type, although former smoking was only associated with increased cancer mortality and not with cardiovascular disease compared to current smoking. Adjustment for blood cadmium concentrations attenuated the association between current smoking and mortality outcomes similarly for smokers of menthol and nonmenthol cigarettes, suggesting that it contributes similarly for both types of cigarette smokers. The findings from the meta-analysis indicate statistically significantly inverse risk for lung cancers and any cancers comparing menthol to nonmenthol cigarette use with no difference for other outcomes, except maybe an increased risk for cardiovascular disease. The interpretation of these associations however is limited by the small number of studies and the heterogeneity across studies regarding study design, outcome definition and follow-up. 

Two studies were also conducted using data from NHANES participants. In 5,167 participants ≥ 20 years of age from NHANES 2001- 2008, menthol cigarette use was cross-sectionally associated with self-reported history of stroke (Odds ratio 2.25, 95% confidence interval 1.33, 3.78) but no difference was observed with self-reported myocardial infarction or other outcomes (hypertension, congestive heart failure or chronic obstructive pulmonary disease) [37]. In our study, we could not evaluate the association with stroke mortality due to the small number of cases. In 5,973 participants ≥ 40 years of age from NHANES 1999-2004, the prevalence of peripheral artery disease (measured ankle-brachial blood pressure index <0.9) was similar comparing menthol and nonmenthol cigarette smokers [36]. 

Several studies have also evaluated differences between menthol and nonmenthol cigarette use and the risk of cardiovascular and cancer endpoints in populations different from NHANES [14,28–30,34,39,40]. In 1535 smokers from Birmingham, AL, Chicago, IL, Minneapolis, MN, and Oakland, CA who participated in the Coronary Artery Risk Development in Young Adults (CARDIA) study, cumulative exposure to menthol cigarettes (per 10-pack year increase) was not associated with the prevalence of coronary artery calcification compared to nonmenthol cigarette use although this study did not report data on non-smokers [14]. In 5,887 smokers with mild lung impairment from Baltimore, MD, Birmingham, AL, Cleveland, OH, Detroit, MI, Los Angeles, CA, Pittsburgh, PA, Portland, OR, Rochester, MN, Salt Lake City, UT and Winnipeg, Canada, the Lung Health Study found no significant difference in risk of coronary heart disease mortality, cardiovascular disease mortality or all-cause mortality over 14 years of follow-up comparing baseline self-reported menthol cigarette use to nonmenthol cigarette use [34]. This study, however, was not a community-based sample and only about 4% of the cohort was African American. 

In the present analysis we found a non-statistically significant inverse risk for total cancer mortality, although we could not evaluate differences in mortality for lung cancer or for other types of cancer due to the small number of cases. Findings from the meta-analysis indicate an inverse cancer risk among menthol cigarette smokers compared to nonmenthol cigarette smokers, particularly for lung cancer. These findings are similar to those from a previous systematic review of menthol cigarette use and lung cancer risk which found an inverse risk for lung cancer among menthol cigarette smokers compared to nonmenthol cigarette smokers, although these findings were statistically significant in females only (Pooled relative risk [95% CI] Overall: 0.93 [0.84, 1.02]; Females: 0.80 [0.67, 0.95]; Males: 1.01 [0.84, 1.22]; Whites: 0.87 [95% CI: 0.75-1.03]; Blacks 0.90 [0.73-1.10])[42]. Overall, the possible inverse association between menthol cigarette use compared to nonmenthol cigarettes for cancer outcomes should be interpreted with caution due to potential limitations in the available studies [43]. Reasons for these inverse associations could be related to the relative short follow-up in these studies. Also information on changes in smoking intensity or cigarette type during follow-up was unavailable which may be important for attributing outcomes to menthol or nonmenthol cigarette use. In a study of 4,832 current smokers who participated in the 1987 National Health Interview Survey Cancer Control Supplement and where followed for 20 years for mortality through linkage with the National Death Index, menthol cigarette use was also associated with a non-statistically significantly inverse risk for lung cancer mortality compared to nonmenthol cigarette use (HR: 0.69, 95% CI: 0.45, 1.06) [35]. On the other hand, in a prospective cohort study of 11,761 members of the Kaiser Permanente Medical Care Program, Northern California Region followed for 12 years, lung cancer risk was increased among male menthol smokers (RR: 1.45, 95% CI 1.03, 2.02) but not among female menthol smokers (RR: 0.75, 95% CI: 0.51, 1.11) [31]. In another prospective study using data from the same program, no differences were observed comparing menthol vs. nonmenthol cigarette use for incident smoking-related cancers different from lung cancer including upper aerodigestive, pancreas, renal adenocarcinoma, other urinary tract, and uterine cervix after 15 years of follow-up [27]. 

### Strengths and Limitations

This study, characterized by rigorous quality control measures, was conducted in a representative sample of the U.S. population and is strengthened by its prospective design. Also, previous studies examining differences in health outcomes associated with menthol cigarette use have been limited to African-American and White smokers; while in our study we also included smokers who were Mexican-American or from other race/ethnicity. The study has some limitations, including a short follow-up time for outcome development and a small number of events. The short follow-up time could affect cancer outcomes more than cardiovascular outcomes, as the latency for cancer development is markedly longer. In sensitivity analyses excluding from the exclusion criteria and analysis covariates that had a significant number of missing values (total cholesterol, HDL cholesterol, cholesterol lowering medication use and eGFR; overall study sample size=10,794, number of all-cause deaths=703), the findings were similar although somewhat attenuated. Also, mortality outcomes were obtained from death certificates. Data from death certificates without confirmation from medical records may be susceptible to miscoding errors in cause of disease. Smoking status and cigarette type were only evaluated at baseline (time of NHANES examination). It is thus possible that participants could have quit smoking or changed their smoking behavior during follow-up, which would likely result in an underestimation of the association between smoking and mortality outcomes, although it is unlikely to be differential by cigarette type. Also, while we had no information on duration of smoking for different types of cigarette or on switching across types, the use of menthol cigarettes has been shown to be relatively constant over time and individuals are unlikely to switch between menthol and nonmenthol [14,34,44]. Information on cigarette type (menthol vs. nonmenthol) was not available for former smokers; therefore, we were unable to assess the influence of cigarette type among former smokers although, as expected, former smoking status was not associated with cardiovascular disease and only cancer mortality remained associated with former smoking status. Finally, there is the potential for unmeasured confounding particularly by other environmental exposures such as secondhand tobacco smoke or air pollution, which are associated with the mortality outcomes in this study.

## Conclusions

Preference for menthol cigarettes may be due to the perception that these cigarettes are less harmful than nonmenthol cigarettes [45–48]. In a representative sample of U.S. adults, current cigarette use was associated with increased all-cause, cardiovascular and cancer mortality compared to never smokers, with no difference comparing menthol and nonmenthol cigarette smokers. Compared to nonmenthol cigarette users, menthol smoking was associated with inverse risk of cancer with some evidence of an increased risk for cardiovascular disease. In vivo and in vitro experiments evaluating potential differences in cardiovascular and cancer effects of menthol and nonmenthol cigarettes are needed to provide mechanistic support for potential epidemiological differences in the health effects of menthol cigarettes. Given the importance of menthol cigarette use and the few available prospective studies investigating cigarette type and disease outcomes, additional prospective studies with longer follow-up are needed to evaluate the relationship between menthol and nonmenthol cigarette use with fatal and non-fatal health outcomes. 

## Supporting Information

Checklist S1 **PRISMA checklist.**
(DOC)
